# Long-term persistent hypertension following surgical resection of pheochromocytoma and paraganglioma

**DOI:** 10.1530/EC-25-0714

**Published:** 2026-03-04

**Authors:** Xingzuo Jiang, Nan Guo, Hao Zhang, Chengyuan Wang, Hongwei Jing, Tao Liu

**Affiliations:** ^1^ Department of Urology, The First Hospital of China Medical University, Shenyang, China; ^2^ Department of Anesthesiology, Shengjing Hospital of China Medical University, Shenyang, China

**Keywords:** long-term persistent hypertension, pheochromocytoma and paraganglioma, follow-up, adrenal gland

## Abstract

**Background:**

Non-metastatic pheochromocytomas and paragangliomas (PPGLs) are considered a curable cause of secondary hypertension. However, some studies identified that a considerable ratio of patients (10.0–43.8%) still experience persistent hypertension even after successful resection of PPGLs.

**Methods:**

We conducted a retrospective analysis of 472 PPGL patients who underwent surgical resection at three centers from January 1, 2012, to October 31, 2022. Comprehensive clinical data were recorded. Binary unconditional logistic analysis was conducted to identify the independent variables associated with long-term persistent hypertension.

**Results:**

The cohort had an average age of 50.5 years, and the median follow-up duration was 61 months. A total of 26.3% of PPGL patients experienced long-term persistent hypertension. After multivariate analysis, we identified older age (odds ratio (OR): 1.021, *P* = 0.008), higher body mass index (BMI, OR: 1.088, *P* = 0.004), lower left ventricular ejection fraction (LVEF, OR: 3.506, *P* = 0.006), and developed intraoperative hemodynamic instability (HDI, OR: 2.053, *P* = 0.002) as independent risk factors for long-term persistent hypertension in PPGL patients.

**Conclusion:**

This study demonstrated that nearly a quarter of PPGL patients still suffer from persistent hypertension after successful resection and identified several risk factors, such as older age, higher BMI, lower LVEF, and developed intraoperative HDI. These results might contribute to improving long-term follow-up strategies.

## Introduction

Pheochromocytomas and paragangliomas (PPGLs) are rare neuroendocrine tumors originating from chromaffin cells in the adrenal medulla and sympathetic/parasympathetic ganglia, respectively (1). Anatomical location is used to distinguish between them. The incidence of PPGLs in the population is in the range of 6.6 cases per million (2). Typical clinical symptoms of PPGLs include paroxysmal or persistent hypertension and the classic triad of headache, palpitations, and profuse sweating (3). Additional symptoms may include pale skin, feelings of panic or anxiety, chest discomfort, nausea, vomiting, weakness, fatigue, weight loss, and constipation (4). Among the clinical symptoms and signs mentioned above, high blood pressure is the classic clinical clue for PPGLs (5). Approximately 65–80% of PPGL patients suffer from hypertension (6).

Surgical resection of PPGLs combined with a preoperative combination of *α*-blockers is an effective treatment option for PPGLs (7). However, previous studies reported that some patients still experienced long-term persistent hypertension even after successful surgical resection of PPGLs. Stenstrom *et al.* reported in a retrospective analysis of 64 patients with successfully resected pheochromocytomas that 18.75% (*n* = 12) patients had long-term persistent hypertension, and the mean follow-up of their study was 12 years (8). In another study retrospectively analyzing 72 patients with successful surgical resection of pheochromocytomas, Modlin *et al.* reported that 33.33% (*n* = 24) patients continued to have long-term persistent hypertension after surgery, and their follow-up lasted at least one year (9). However, these studies were published in the last century with a small sample size from one center and did not include patients with paragangliomas. Recognizing the risk of recurrence and long-term sequelae, contemporary clinical guidelines emphasize a structured long-term follow-up for all PPGL patients. The European Society of Endocrinology (ESE) clinical practice guidelines, for instance, recommend that all patients operated on for PPGLs should be followed up for at least 10 years, with lifelong annual follow-up advised specifically for high-risk patients, such as those who are young and have a genetic predisposition, large tumors, and/or paragangliomas (10). While these guidelines provide a crucial framework, the stratification of ‘high risk’ is primarily based on tumor genetics and anatomy. Risk factors for a distinct long-term outcome – persistent hypertension – remain poorly defined. Therefore, based on this large multi-center cohort, we aimed not only to determine the incidence of long-term persistent hypertension after PPGL resection but also to identify novel and modifiable patient-specific risk factors associated with this condition. Our findings may refine follow-up strategies by identifying a broader subgroup of patients who might benefit from intensified, potentially lifelong, surveillance.

## Materials and methods

### Study design and participants

Our retrospective analysis covered 979 consecutive patients who underwent surgical resection of PPGLs across three centers: (1) Nanhu center at Shengjing Hospital of China Medical University, (2) The First Affiliated Hospital of China Medical University, and (3) Huaxiang center at Shengjing Hospital of China Medical University, from January 1, 2012, to October 31, 2022. All patients had a minimum follow-up period of one year.

### Inclusion and exclusion criteria

**Figure 1:**
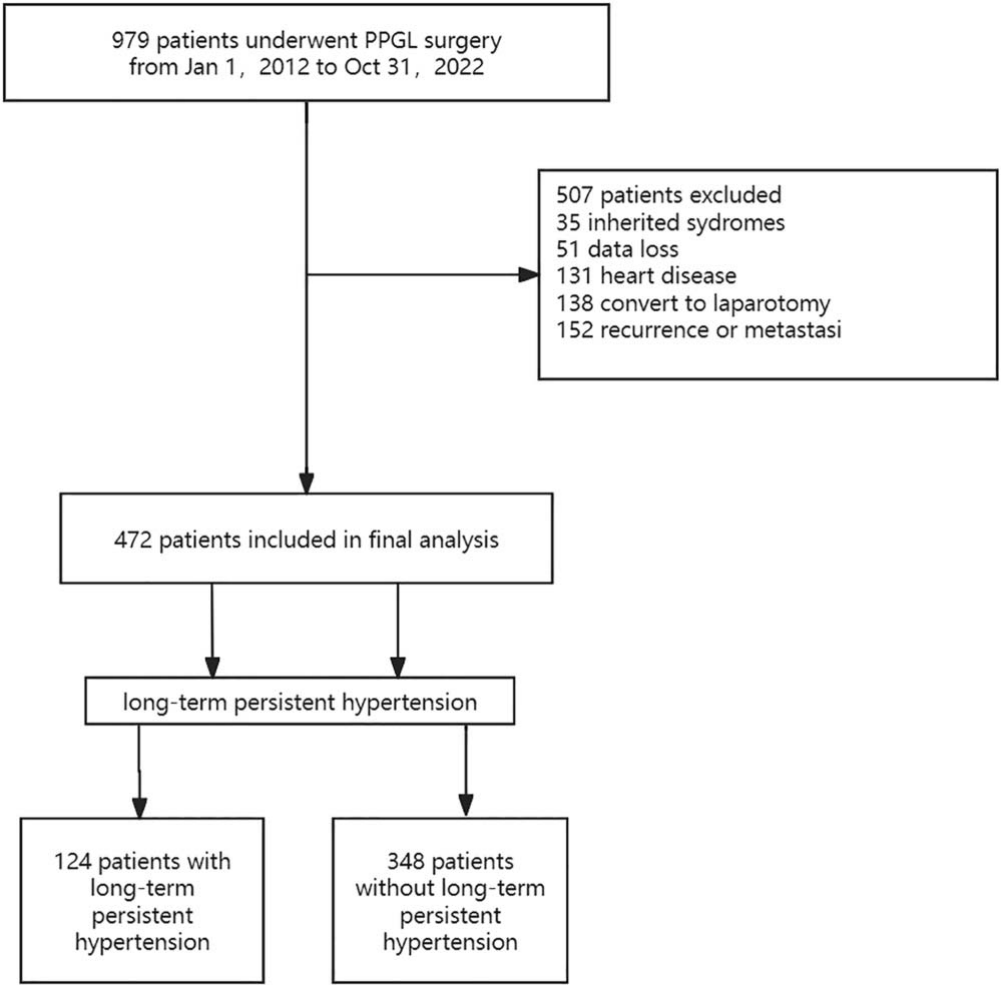
Process of patient selection.

Patients diagnosed with PPGLs were confirmed through pathological examination. All included patients had preoperative hypertension. The study included those who underwent either unilateral laparoscopic or open tumor resection. These patients were in the clinical stage of localized disease and had an American Society of Anesthesiologists (ASA) score ranging from 1 to 3 (11). Exclusion criteria were applied to construct a homogeneous cohort of patients with definitively cured, localized PPGLs, with the aim of isolating risk factors for hypertension as a tumor sequela rather than those confounded by ongoing disease or complex management. In particular, we excluded: i) patients who required conversion to laparotomy, as these cases were associated with intraoperative injuries that could independently influence outcomes; ii) those with a history of congenital heart disease, cardiac surgery, severe heart valve disease, or cardiac amyloidosis, to avoid confounding by primary cardiovascular pathology; iii) those affected by inherited syndromes (MEN2A/VHL/NF) or multi-focal tumors, due to their distinct biology and lifelong risk of new tumors, which present profound confounding factors; and iv) patients presenting tumor recurrence or metastasis at follow-up, since persistent disease is a direct and predominant cause of hypertension that would obscure the subtler effects of a cured state. Following these criteria, 472 patients were deemed eligible for the study. The process of patient selection is detailed in Fig. 1.

### Interventions

Patients exhibiting typical biochemical and radiographic indications of PPGLs underwent comprehensive preoperative medical preparation. This regimen involved administering selective or non-selective *α*-blockers for at least two weeks, supplemented by *β*-blockers for tachycardia and calcium channel blockers when necessary. Exceptions were made for patients presenting normal biochemical tests coupled with atypical radiographic signs of a PPGL. The protocol also recommended a high-sodium diet and increased fluid intake to counteract the blood volume contraction caused by catecholamines. The criteria for adequate preoperative preparation were a blood pressure below 130/80 mmHg and a heart rate under 90 beats per minute. All patients received general anesthesia, and the surgical procedures were carried out by experienced surgeons and anesthesiologists, ensuring high clinical proficiency.

### Definition of successful resection and cure

Successful resection and biochemical cure of PPGLs were defined by meeting both of the following criteria postoperatively:

Biochemical normalization: Urinary levels of catecholamines and metanephrines (in particular, the 24-h urinary VMA, MN, and NMN levels used in our centers) returned to within the normal reference range on follow-up testing, typically performed 6–12 months after surgery and in the absence of confounding medications.

Radiologic confirmation: No evidence of residual tumor, local recurrence, or metastasis was found on cross-sectional imaging (computed tomography) performed 3–6 months postoperatively and on all subsequent surveillance scans throughout the follow-up period.

### Follow-up

In alignment with established guidelines, long-term postoperative monitoring of patients is advised (12). We suggest conducting evaluations every six months during the initial two years, followed by annual assessments thereafter (13). Our review encompassed electronic medical records from the three involved centers, focusing on patient symptoms, signs, blood pressure, urine catecholamine levels, and imaging studies to assess for tumor recurrence or metastasis. In October 2022, a comprehensive telephone survey was conducted with all participating patients. During this interview, patients provided updates on their current health status, ongoing medications, and any new-onset cardiovascular complications. Medical records from the hospitals were used to corroborate all self-reported cardiovascular complications.

### Outcomes

We recorded patient demographics, including age, sex, body mass index (BMI), ASA scores, and comorbidities, such as diabetes mellitus and coronary artery disease (CAD) or stroke. In addition, family history of hypertension, PPGL classification, and preoperative data, such as tumor size, 24-h urine VMA/MN/NMN levels, and LVEF, were considered. Intraoperative details, including the surgical approach, instances of HDI, surgery duration, and blood loss, were also captured. Short-term postoperative data focused on cardiovascular complications and hospital stay length, while long-term follow-up data encompassed the duration, persistent hypertension, and long-term cardiovascular complications.

Hypertension was defined as a blood pressure higher than 140/90 mmHg measured on three separate occasions in a quiet and suitable environment after admission to hospital with adequate rest or ongoing use of antihypertensive medication. For blood pressure monitoring of follow-up patients, all patients are advised to process in-office assessments, or if they are unable to have their blood pressure measured at the clinic, they are advised to self-monitor at home using an electronic sphygmomanometer. They were advised to:

i) Use a validated, automatic upper-arm cuff sphygmomanometer.
ii) Measure BP in a seated position after 5 min of rest.
iii) Take duplicate readings, 1–2 min apart, in the morning and evening.
iv) Record measurements for at least 3 consecutive days.

Low LVEF was less than 50%, indicating left ventricular systolic dysfunction (14). Cardiovascular complications were broadly defined to include incidences or deaths caused by CAD, myocardial infarction, arrhythmia, heart failure, stroke, and pulmonary embolism or deep vein thrombosis (15, 16). Long-term cardiovascular complications were specified as new-onset issues emerging at least one-year post-surgery for PPGLs. Intraoperative HDI was identified by extremely high or low blood pressure during surgery, defined as a systolic blood pressure (SBP) ≥ 200 mmHg or <80 mmHg or mean arterial pressure <60 mmHg (17, 18). Persistent hypertension post-surgery was defined as a sustained blood pressure of ≥140/90 mmHg during follow-up. Importantly, all patients who required continuous antihypertensive therapy to maintain blood pressure below this threshold were classified as hypertensive, irrespective of their preoperative antihypertensive regimen.

### Statistical analysis

Statistical analyses in this study were conducted using SPSS, version 26.0. We presented continuous variables as means with standard deviations, medians, or interquartile ranges, depending on their distribution. Categorical variables were shown as counts and percentages. The normality of the data was assessed using the Kolmogorov–Smirnov test, with normally distributed data presented as mean ± standard deviation (SD) and non-normally distributed (nonparametric) data as medians with interquartile ranges (IQRs). We employed the chi-square test for categorical variable comparisons, while continuous variables were compared using either the *t*-test or the Mann–Whitney *U* test based on their distribution. To identify independent risk factors, a multivariate binary logistic regression analysis was performed. Variables with a *P*-value < 0.10 in the univariate analysis were eligible for inclusion in the initial model. A backward stepwise selection procedure (conditional likelihood ratio) was employed to derive the final parsimonious model, with a variable removal threshold set at *P* > 0.10. Prior to model fitting, multicollinearity among candidate predictors was assessed by calculating variance inflation factors (VIFs) in a preparatory linear regression analysis. Furthermore, the linearity assumption of continuous variables (age and BMI) on the logit scale was evaluated using restricted cubic splines with 4 knots and compared to linear models via likelihood ratio tests. Statistical significance was established at a two-tailed *P* value of less than 0.05.

## Results

This study included 472 patients (pheochromocytoma 370/paraganglioma 102) based on our inclusion and exclusion criteria. The median follow-up duration was 61 months. The cohort had an average age of 50.5 years, with a nearly equal male-to-female ratio (F/M: 54.7%/46.3%) and an average BMI of 23.1 kg/m^2^. During the follow-up period, 26.3% (124/472) patients continued to experience long-term persistent hypertension. Detailed demographic data of the patients are presented in Table 1.

**Table 1:** Demographics and clinical data in this cohort and univariate analysis according to long-term persistent hypertension in PPGL patients after successful surgery.

	All patients (%) *n* = 472 (100)	Long-term persistent hypertension		*P* value
		No (%) *n* = 348 (73.7)	Yes (%) *n* = 124 (26.3)	
Follow-up time (months)	61 (42, 78)	60.5 (42.0, 76.0)	62.0 (42.0, 79.0)	0.451
Demographic characteristics				
Age (years)	50.5 ± 15.3	49.4 ± 15.9	53.4 ± 13.0	0.007
Sex (F/M)	258 (54.7)/214 (45.3)	192 (55.2)/156 (44.8)	66 (53.2)/58 (46.8)	0.708
BMI (kg/m^2^)	23.1 ± 3.9	22.8 ± 4.2	24.0 ± 2.5	<0.001
ASA (scores)	2 (2.3)	2 (2.3)	2 (2.3)	0.733
Comorbidities				
Diabetes mellitus (yes)	96 (20.3)	66 (19.0)	30 (24.2)	0.214
CAD/stroke (yes)	76 (16.1)	50 (14.4)	26 (21.0)	0.086
Family history of hypertension (yes)	18 (3.8)	14 (4.0)	4 (3.2)	0.901
Classification of PPGLs				
Pheochromocytoma/paraganglioma	370 (78.4)/102 (21.6)	272 (78.2)/76 (21.8)	98 (79.0)/26 (21.0)	0.84
Preoperative data				
Urinary VMA or MN or NMN/normal upper limit	2.2 ± 1.6	2.2 ± 1.7	2.1 ± 1.6	0.677
Tumor size (cm)	4.9 ± 2.3	5.0 ± 2.3	4.7 ± 2.3	0.193
LVEF (low)	22 (4.7)	10 (2.9)	12 (9.7)	0.002
Intraoperative data				
Laparoscopy/open	348 (73.7)/124 (26.3)	262 (75.3)/86 (24.7)	86 (69.4)/38 (30.6)	0.197
HDI (yes)	154 (32.6)	102 (29.3)	52 (41.9)	0.010
Surgery duration (mins)	160 (120.3, 208)	164.5 (125, 214)	149 (99, 184)	0.008
Estimated blood loss (mL)	100 (50, 200)	100 (50, 200)	100 (50, 200)	0.737
Short-term postoperative data				
Cardiovascular complications (yes)	20 (4.2)	16 (4.6)	4 (3.2)	0.515
Length of stay (days)	9.6 ± 4.1	9.7 ± 4.2	9.3 ± 3.5	0.424

Continuous variables were expressed as median (interquartile range) or mean ± standard deviation; categorical variables were reported as number (percentage). Independent-samples Student’s *t*-test was used to compare means of two continuous normally distributed variables, and the Mann–Whitney *U* test was run to determine the mean of two continuous non-normally distributed variables. Number was provided for a categorical variable (percentage). The chi-squared test and Fisher’s exact test were used to compare categorical variables.

Abbreviations: F, female; M, male; BMI, body mass index; ASA, American Society of Anesthesiologists; CAD, coronary artery disease; PPGLs, pheochromocytomas and paragangliomas; VMA, vanillylmandelic acid; MN, metanephrine; NMN, normetanephrine; LVEF, left ventricular ejection fraction; and HDI, hemodynamic instability.

Based on univariate analysis, we found that older age (49.4 vs 53.4 years, *P* = 0.007) and higher BMI (22.8 kg/m^2^ vs 24.0 kg/m^2^, *P* < 0.001) were more prone to long-term persistent hypertension. Shorter surgery duration (164.5 vs 149 min, *P* = 0.008) was also considered to be associated with long-term persistent hypertension. In addition, we observed that a higher proportion of patients with long-term persistent hypertension experienced CAD/stroke (14.4 vs 21.0%, *P* = 0.086), HDI (29.3 vs 41.9%, *P* = 0.010), and reduced LVEF (2.9 vs 9.7%, *P* = 0.001). These variables were also deemed related to long-term persistent hypertension.

We selected variables with *P*-values less than 0.10 for the binary logistic analysis or that were clinically relevant from Table 1. This analysis revealed that older age (odds ratio (OR): 1.021, 95% confidence interval (CI): 1.006–1.036, *P* = 0.008), higher BMI (OR: 1.088, 95% CI: 1.027–1.152, *P* = 0.004), lower LVEF (OR: 3.506, 95% CI: 1.433–8.575, *P* = 0.006), and developed intraoperative HDI (OR: 2.053, 95% CI: 1.305–3.230, *P* = 0.002) were independent risk factors for long-term persistent hypertension. These results are detailed in Table 2.

**Table 2:** Multivariate logistic regression of long-term persistent hypertension.

Variable	*β*	OR	95% CI	*P*
Age (years)	0.021	1.021	1.006–1.036	0.008
BMI (kg/m^2^)	0.084	1.088	1.027–1.152	0.004
LVEF (low)	1.254	3.506	1.433–8.575	0.006
HDI (yes)	0.719	2.053	1.305–3.230	0.002
Surgery duration (minutes)	−0.001	0.999	0.996–1.002	0.529
CAD/stroke (yes)	0.346	1.413	0.790–2.521	0.232

The *β* coefficient, odds ratio, and 95% confidence interval were measured through binary logistic regression.

Abbreviations: BMI, body mass index; CAD, coronary artery disease; LVEF, left ventricular ejection fraction; HDI, hemodynamic instability.

## Discussion

While several previous studies have documented cases of long-term persistent hypertension following PPGL surgery, there remains a lack of research exploring the determinants of this specific hypertension condition. Therefore, we conducted a multi-center retrospective analysis. This study indicated that older age, higher BMI, lower LVEF, and developed intraoperative HDI were significantly associated with long-term persistent hypertension in PPGL patients after successful surgery.

Prior studies have shown that the incidence of long-term persistent hypertension following PPGL surgery ranges from 10.0 to 43.8% (8, 19, 20, 21). In this study, we observed a 26.3% incidence of long-term persistent hypertension, which aligns well with the reported range in the existing literature. For details, please see Supplementary Table 1 (see section on Supplementary materials given at the end of the article).

This study demonstrated that patients with older age were more likely to have long-term persistent hypertension. Stenstrom *et al.*, in a retrospective analysis of 64 patients with pheochromocytoma, found that the mean age of the patients in the long-term persistent hypertension group (*n* = 28) was higher than the mean age of the normal group (45.1 vs 40.1 years) (8). In addition, Paolo *et al.*, in a study that retrospectively analyzed 48 patients with functional adrenal tumors, identified age as the only risk factor for long-term persistent hypertension (22). In another study prospectively examining 26 patients undergoing successful pheochromocytoma resection, Pradeep *et al.* noted that older patients were more likely to have long-term persistent hypertension (23). One possible reason for this is a decrease in nitric oxide and the production of endothelium-dependent contractile factors due to aging, which leads to endothelial dysfunction (24). Moreover, endothelial dysfunction is thought to be strongly associated with the development of persistent hypertension (25).

This study also indicated that patients with higher BMI were more likely to develop long-term persistent hypertension after undergoing PPGL surgery. The research conducted by Pradeep *et al.* also explored the association between BMI and the development of hypertension following pheochromocytoma surgery. However, they found no statistically significant relationship (*P* = 0.50) (23). This lack of significance is likely due to the small sample size of their study (*n* = 24), which exclusively involved patients with pheochromocytomas. In addition, the follow-up period was limited to just three months post-surgery. This short follow-up duration impacted the ability to assess long-term hypertension outcomes accurately. In our study, the median BMI of patients with persistent hypertension was 24.0 (kg/m^2^), which is very close to the BMI criteria for overweight or obesity (BMI ≥25.0 kg/m^2^). It is commonly estimated that at least 75% of the development of hypertension is associated with obesity (26). Obesity affects the development of hypertension through several mechanisms, such as increased sympathetic nervous system activity in obese patients, which promotes vasoconstriction and increases peripheral vascular resistance, leading to hypertension (27). Considering that the prevalence of hypertension and obesity increases with age according to the available epidemiological data (28), we should pay more attention to those patients who are older and have higher BMI in the follow-up visits.

PPGLs release catecholamines, which directly affect the myocardium through mechanisms such as calcium overload, changes in cell membrane permeability, and increased lipid mobility, which may lead to decreased LVEF (29). Our study found a significant association between LVEF below 50% and long-term persistent hypertension (*P* = 0.006). On the contrary, a clinical trial by Dirk *et al.* comparing normal patients, hypertensive patients, and patients with PPGLs noted that the LVEF of patients with PPGLs was essentially similar to that of normal patients (67 ± 1 vs 68 ± 1) (30). However, their study had a small sample size and did not differentiate between patients with long-standing persistent hypertension. Although previous studies have shown a negative correlation between changes in LVEF and changes in mean blood pressure and diastolic blood pressure (31), the association between LVEF and hypertension still requires further study.

Most studies on HDI in PPGLs have focused on predicting risk factors for its occurrence (17, 32, 33, 34, 35, 36). This study first found the association between HDI and long-term persistent hypertension in PPGL patients after surgery. In a retrospective analysis of 114 patients who developed HDI during surgical resection of PPGLs, Jung *et al.* identified higher urinary epinephrine levels as a potential causative factor (37). This observation is particularly relevant considering the cardiotoxic effects associated with elevated catecholamine doses, which can lead to myocardial fibrosis, a form of irreversible cardiac damage (38). Such a cardiac impairment can significantly disrupt the heart’s ability to regulate blood pressure effectively. In conclusion, further, more robust evidence is needed in the future to demonstrate the association between HDI and long-term persistent hypertension.

This study has certain limitations. First, our data came from retrospective data from three centers, and standards such as preoperative tests needed to be more consistent across centers. Second, due to the long recruitment period, perioperative medical preparation changes may have affected the results. Third, some other potential factors, such as genetic mutations, were not included because about 40% of PPGLs occurs in autosomal dominant familial disorders (39). Therefore, these results need to be validated in other cohorts. Fourth, due to the limitations of the retrospective study in this paper, we were only able to ask patients about their postoperative blood pressure by telephone follow-up, and we were not able to have all patients undergo in-office blood pressure measurements, which may have had an impact on the rate of postoperative hypertension. Fifth, due to the inability to measure plasma-free metanephrines in our center, we applied 24-h urine fractionated metanephrines (MN/NMN) and VMA levels as the primary biochemical diagnostic and follow-up markers; however, VMA measurement is no longer recommended by the guidelines (5). Future relevant studies could focus on the effect of plasma metanephrines on long-term persistent hypertension. In addition, due to the retrospective nature of our study, we did not systematically collect detailed preoperative and postoperative systolic and diastolic blood pressure values. The lack of continuous blood pressure data may limit a more granular analysis of blood pressure trends and severity in this cohort. Future prospective studies should include structured blood pressure profiling to further characterize cardiovascular outcomes after PPGL resection. In conclusion, within the specific context of patients with successfully resected, sporadic, localized PPGLs, we have identified several clinical predictors of long-term persistent hypertension. While these factors provide valuable guidance for post-operative follow-up in this defined group, their applicability to patients with hereditary syndromes, multi-focal, or non-curative disease requires further investigation, as the pathophysiology of hypertension in those contexts may be substantially different. Nevertheless, this study is the first large multi-center study to analyze risk factors in a PPGL cohort. It may help improve long-term follow-up strategies.

## Conclusion

In summary, even after successful PPGL resection, a clinically significant proportion of patients (26.3% in our cohort) remain hypertensive. This indicates that cure of the tumor does not guarantee normalization of blood pressure in all patients, warranting continued vigilance and long-term follow-up, especially in those with the identified risk factors (older age, higher BMI, lower LVEF, and HDI).

## Supplementary materials



## Declaration of interest

The authors certify that there is no conflict of interest, including any specific financial interest or relationship or affiliation relevant to the subject matter or materials discussed in the manuscript (e.g., employment/affiliation, grants or funding, consultancies, honoraria, stock ownership or options, expert testimony, royalties, or patents filed, received, or pending).

## Funding

This work was supported by the Shenyang Science and Technology Plan Project (Grant No.24-214-3-21), and the Science and Technology Planning Project of Liaoning Province of China (Grant No. 2023JH2/20200090).

## Author contribution statement

Hongwei Jing and Tao Liu had full access to all the data in the study. They took responsibility for the data’s integrity and the data analysis’s accuracy. Hongwei Jing and Tao Liu developed the protocol/project. Xingzuo Jiang and Nan Guo collected and managed the data. Xingzuo Jiang and Nan Guo analyzed the data. Xingzuo Jiang, Nan Guo, Hao Zhang, and Chengyuan Wang wrote or edited the manuscript.

## Data availability

The datasets generated and/or analyzed during the current study are not publicly available because the dataset contains sensitive information related to personal privacy and data protection regulations. To ensure the protection of participants’ privacy and compliance with relevant laws and regulations, the authors must strictly manage the data. However, they are available from the corresponding author on reasonable request.

## Ethical statement

The Ethics Committee of Shengjing Hospital Affiliated China Medical University provided ethical approval (2018 PS398K). The study protocol conformed to the ethical guidelines of the 1975 Declaration of Helsinki.

## Waiver of informed consent for retrospective study statement

Given the retrospective nature of our study, and in accordance with the guidelines and requirements set forth by the Shengjing Hospital of China Medical University Ethics Committee, an informed consent waiver has been granted. This decision is based on the assessment that the research involves no more than minimal risk to participants and involves the use of existing data, documents, records, or specimens that have been collected for non-research purposes. The confidentiality of all personal information will be strictly maintained, and all data used in this study will be anonymized to protect participant privacy. The Shengjing Hospital of China Medical University Ethics Committee has reviewed and approved the research protocol, including the waiver of informed consent, recognizing that the study’s retrospective design makes it impracticable to obtain consent from all participants whose data are being analyzed. This waiver does not diminish the ethical standards to which this research is held; all efforts will be made to ensure the integrity and confidentiality of the data. This declaration is to confirm that our manuscript acknowledges the waiver of informed consent for our study, as approved by the Shengjing Hospital of China Medical University Ethics Committee, and to affirm our commitment to conducting this research with the utmost respect for ethical standards and participant privacy.

## References

[1] Buffet A, Burnichon N, Favier J, et al. An overview of 20 years of genetic studies in pheochromocytoma and paraganglioma. Best Pract Res Clin Endocrinol Metab 2020 34 101416. (10.1016/j.beem.2020.101416) 32295730

[2] Leung AA, Pasieka JL, Hyrcza MD, et al. Epidemiology of pheochromocytoma and paraganglioma: population-based cohort study. Eur J Endocrinol 2021 184 19–28. (10.1530/eje-20-0628) 33112261

[3] Fang F, Ding L, He Q, et al. Preoperative management of pheochromocytoma and paraganglioma. Front Endocrinol 2020 11 586795. (10.3389/fendo.2020.586795) PMC7551102 33117294

[4] Geroula A, Deutschbein T, Langton K, et al. Pheochromocytoma and paraganglioma: clinical feature-based disease probability in relation to catecholamine biochemistry and reason for disease suspicion. Eur J Endocrinol 2019 181 409–420. (10.1530/eje-19-0159) 31370000

[5] Lenders JWM, Kerstens MN, Amar L, et al. Genetics, diagnosis, management and future directions of research of phaeochromocytoma and paraganglioma: a position statement and consensus of the working group on Endocrine Hypertension of the European Society of Hypertension. J Hypertens 2020 38 1443–1456. (10.1097/hjh.0000000000002438) 32412940 PMC7486815

[6] Kiriakopoulos A, Giannakis P & Menenakos E. Pheochromocytoma: a changing perspective and current concepts. Ther Adv Endocrinol Metab 2023 14 20420188231207544. (10.1177/20420188231207544) 37916027 PMC10617285

[7] Neumann HPH, Young WF Jr. & Eng C. Pheochromocytoma and paraganglioma. N Engl J Med 2019 381 552–565. (10.1056/nejmra1806651) 31390501

[8] Stenstrom G, Ernest I & Tisell LE. Long-term results in 64 patients operated upon for pheochromocytoma. Acta Med Scand 1988 223 345–352. (10.1111/j.0954-6820.1988.tb15883.x) 3369315

[9] Modlin IM, Farndon JR, Shepherd A, et al. Phaeochromocytomas in 72 patients: clinical and diagnostic features, treatment and long term results. Br J Surg 1979 66 456–465. (10.1002/bjs.1800660704) 466037

[10] Plouin PF, Amar L, Dekkers OM, et al. European society of endocrinology clinical practice guideline for long-term follow-up of patients operated on for a phaeochromocytoma or a paraganglioma. Eur J Endocrinol 2016 174 G1–G10. (10.1530/eje-16-0033) 27048283

[11] Horvath B, Kloesel B, Todd MM, et al. The evolution, current value, and future of the American Society of Anesthesiologists physical status classification system. Anesthesiology 2021 135 904–919. (10.1097/aln.0000000000003947) 34491303

[12] Kumar RM, Violette PD, Tran C, et al. Canadian Urological Association Best Practice Report: long-term surveillance following resection of pheochromocytoma. Can Urol Assoc J 2019 13 372–376. (10.5489/cuaj.6254) 31799918 PMC6892687

[13] Conzo G, Musella M, Corcione F, et al. Laparoscopic adrenalectomy, a safe procedure for pheochromocytoma. A retrospective review of clinical series. Int J Surg 2013 11 152–156. (10.1016/j.ijsu.2012.12.007) 23267853

[14] Yu Y, Chen C, Han W, et al. Metanephrine and normetanephrine associated with subclinical myocardial injuries in pheochromocytoma and paraganglioma. Front Oncol 2022 12 1024342. (10.3389/fonc.2022.1024342) 36237312 PMC9552905

[15] Buscemi S, Di Buono G, D'Andrea R, et al. Perioperative management of pheochromocytoma: from a dogmatic to a tailored approach. J Clin Med 2021 10 3759. (10.3390/jcm10163759) 34442056 PMC8397195

[16] Groeben H, Walz MK, Nottebaum BJ, et al. International multicentre review of perioperative management and outcome for catecholamine-producing tumours. Br J Surg 2020 107 e170–e178. (10.1002/bjs.11378) 31903598 PMC8046358

[17] Takeda T, Hakozaki K, Yanai Y, et al. Risk factors for haemodynamic instability and its prolongation during laparoscopic adrenalectomy for pheochromocytoma. Clin Endocrinol 2021 95 716–726. (10.1111/cen.14557) 34288003

[18] Wang J, Liu Q, Jiang S, et al. Preoperative alpha-blockade versus no blockade for pheochromocytoma-paraganglioma patients undergoing surgery: a systematic review and updated meta-analysis. Int J Surg 2023 109 1470–1480. (10.1097/js9.0000000000000390) 37037514 PMC10389437

[19] Castilho LN, Simoes FA, Santos AM, et al. Pheochromocytoma: a long-term follow-up of 24 patients undergoing laparoscopic adrenalectomy. Int Braz J Urol 2009 35 24–35. (10.1590/s1677-55382009000100005) 19254395

[20] Mishra A, Mehrotra PK, Agarwal G, et al. Pediatric and adolescent pheochromocytoma: clinical presentation and outcome of surgery. Indian Pediatr 2014 51 299–302. (10.1007/s13312-014-0397-9) 24825268

[21] Erickson D, Kudva YC, Ebersold MJ, et al. Benign paragangliomas: clinical presentation and treatment outcomes in 236 patients. J Clin Endocrinol Metab 2001 86 5210–5216. (10.1210/jcem.86.11.8034) 11701678

[22] Sapienza P & Cavallaro A. Persistent hypertension after removal of adrenal tumours. Eur J Surg 1999 165 187–192. (10.1080/110241599750007027) 10231649

[23] Prakash P, Ramachandran R, Tandon N, et al. Changes in blood pressure, blood sugar, and quality of life in patients undergoing pheochromocytoma surgery: a prospective cohort study. Indian J Urol 2019 35 34–40. (10.4103/iju.IJU_190_18) 30692722 PMC6334590

[24] Camici GG, Sudano I, Noll G, et al. Molecular pathways of aging and hypertension. Curr Opin Nephrol Hypertens 2009 18 134–137. (10.1097/mnh.0b013e328326093f) 19434051

[25] Konukoglu D & Uzun H. Endothelial dysfunction and hypertension. Adv Exp Med Biol 2017 956 511–540. (10.1007/5584_2016_90) 28035582

[26] Lloyd-Jones D, Adams R, Carnethon M, et al. Heart disease and stroke statistics--2009 update: a report from the American Heart Association Statistics Committee and Stroke Statistics Subcommittee. J Circ 2009 119 e21–e181. (10.1161/circulationaha.108.191261) 19075105

[27] Leggio M, Lombardi M, Caldarone E, et al. The relationship between obesity and hypertension: an updated comprehensive overview on vicious twins. Hypertens Res 2017 40 947–963. (10.1038/hr.2017.75) 28978986

[28] Mancia G, Kreutz R, Brunstrom M, et al. 2023 ESH guidelines for the management of arterial hypertension the task force for the management of arterial hypertension of the European Society of Hypertension: endorsed by the International Society of Hypertension (ISH) and the European Renal Association (ERA). J Hypertens 2023 41 1874–2071. (10.1097/hjh.0000000000003480) 37345492

[29] Wang C, Yu Y & Yang Y. Correlation between catecholamines and echocardiographic parameters in patients with pheochromocytoma and paraganglioma. J Clin Ultrasound 2023 51 31–35. (10.1002/jcu.23290) 36054716 PMC10087801

[30] Weismann D, Liu D, Bergen T, et al. Hypertension and hypertensive cardiomyopathy in patients with a relapse-free history of phaeochromocytoma. Clin Endocrinol 2015 82 188–196. (10.1111/cen.12536) 25040503

[31] Mandry D, Girerd N, Lamiral Z, et al. Relationship between left ventricular ejection fraction variation and systemic vascular resistance: a prospective cardiovascular magnetic resonance study. Front Cardiovasc Med 2021 8 803567. (10.3389/fcvm.2021.803567) 35004914 PMC8739894

[32] Pang Y, Li M, Jiang J, et al. Impact of body composition and genotype on haemodynamics during surgery for pheochromocytoma and paraganglioma. J Cachexia Sarcopenia Muscle 2022 13 2843–2853. (10.1002/jcsm.13071) 36068986 PMC9745493

[33] Tariel F, Dourmap C, Prudhomme T, et al. Adrenalectomy for pheochromocytoma: complications and predictive factors of intraoperative hemodynamic instability. Am Surg 2023 89 4772–4779. (10.1177/00031348221135774) 36302517

[34] Thompson JP, Bennett D, Hodson J, et al. Incidence, risk factors and clinical significance of postoperative haemodynamic instability after adrenalectomy for phaeochromocytoma. Gland Surg 2019 8 729–739. (10.21037/gs.2019.11.22) 32042681 PMC6989915

[35] Kiernan CM, Du L, Chen X, et al. Predictors of hemodynamic instability during surgery for pheochromocytoma. Ann Surg Oncol 2014 21 3865–3871. (10.1245/s10434-014-3847-7) 24939623 PMC4192065

[36] Li S, Li Z, Zheng J, et al. Risk factors and a predictive nomogram for hemodynamic instability during adrenalectomy for large pheochromocytomas and paragangliomas: a retrospective cohort study. Eur J Surg Oncol 2023 49 106964. (10.1016/j.ejso.2023.06.016) 37369608

[37] Kim JH, Lee HC, Kim SJ, et al. Perioperative hemodynamic instability in pheochromocytoma and sympathetic paraganglioma patients. Sci Rep 2021 11 18574. (10.1038/s41598-021-97964-3) 34535733 PMC8448751

[38] Liaudet L, Calderari B & Pacher P. Pathophysiological mechanisms of catecholamine and cocaine-mediated cardiotoxicity. Heart Fail Rev 2014 19 815–824. (10.1007/s10741-014-9418-y) 24398587

[39] Cornu E, Belmihoub I, Burnichon N, et al. [Phaeochromocytoma and paraganglioma]. Rev Med Interne 2019 40 733–741. (10.1016/j.revmed.2019.07.008) 31493938

