# Supplementary material for: Long-term persistent hypertension following surgical resection of pheochromocytoma and paraganglioma
Source: Endocr Connect. 2026 Mar 4;15(3):e250714. doi: 10.1530/EC-25-0714 (PMC12989711; doi:10.1530/EC-25-0714)
Supplement: Supplementary file 1 [file supplementary_materials.pdf]

Supplementary table 1.Summary and Comparison of the Literature on the Incidence of Persistent Hypertension After Pheochromocytoma/Paraganglioma Resection.

Author, Year	Study Design	Patients (n)	Population	Median/Mean Follow-up	Rate of Persistent Hypertension
Modlin et al., 1979 [9]	Retrospective, SC	72	Pheochromocytoma only	≥12 months	33.3% (24/72)
Stenström et al., 1988 [8]	Retrospective, SC	64	Pheochromocytoma only	~12 years	18.8% (12/64)
Erickson et al., 2001 [19]	Retrospective, SC	236	Paraganglioma only (Benign)	7.4 years	29.2% (69/236)
Castilho et al., 2009 [17]	Retrospective, SC	24	Pheochromocytoma (Laparoscopic)	43 months	41.7% (10/24)
Prakash et al., 2019 [21]	Prospective, SC	26	Pheochromocytoma only	3 months	19.2% (5/26)
Sapienza et al., 1999 [20]	Retrospective, SC	48	Functional adrenal tumors	Not specified	Not specified (but identified age as risk factor)
Present Study (Jiang et al., 2025)	Retrospective, MC	472	Pheochromocytoma & Paraganglioma	61 months	26.3% (124/472)

Abbreviations: SC, Single-Center; MC, Multi-Center.
